# Imaging P‐Glycoprotein Function at the Blood–Brain Barrier as a Determinant of the Variability in Response to Central Nervous System Drugs

**DOI:** 10.1002/cpt.1402

**Published:** 2019-03-23

**Authors:** Martin Bauer, Nicolas Tournier, Oliver Langer

**Affiliations:** ^1^ Department of Clinical Pharmacology Medical University of Vienna Vienna Austria; ^2^ UMR 1023 IMIV Service Hospitalier Frédéric Joliot CEA Inserm Université Paris Sud CNRS Université Paris‐Saclay Orsay France; ^3^ Preclinical Molecular Imaging AIT Austrian Institute of Technology GmbH Seibersdorf Austria; ^4^ Division of Nuclear Medicine Department of Biomedical Imaging and Image‐Guided Therapy Medical University of Vienna Vienna Austria

**P‐glycoprotein (P‐gp) is an efflux transporter expressed at the blood–brain barrier (BBB), which restricts the brain distribution of many drugs. Variability in P‐gp function at the BBB may lead to variability in response to central nervous system (CNS)‐acting drugs and/or CNS adverse effects. Positron emission tomography (PET) with radiolabeled P‐gp substrates has opened up the possibility to directly study P‐gp function at the human BBB. Yet, the role of P‐gp in governing the response to CNS drugs has remained elusive so far.**

There is a large inter individual variability in the response to CNS‐acting drugs, which complicates the treatment of chronic neuropsychiatric diseases. The sources of this variability remain poorly understood, and research has so far mainly focused on pharmacodynamic factors (e.g., disease‐induced or drug‐induced modulation of the affinity, density and availability of CNS targets and their transduction pathways). Pharmacokinetic variability, as a result of, for example, genetic polymorphisms, pathophysiological changes, or peripheral drug–drug interactions (DDIs), has predominantly been explored by the determination of drugs and drug metabolites in plasma and body fluids. However, plasma pharmacokinetics do not generally predict brain exposure and the ability of compounds to reach their CNS targets, mainly due to the presence of the BBB, the main interface between the circulation and brain parenchyma.

The seminal work by Alfred H. Schinkel and colleagues has highlighted the importance of the adenosine triphosphate‐binding cassette (ABC) transporter P‐gp (encoded in humans by the *ABCB1* gene and in rodents by the *Abcb1a* and *Abcb1b* genes) in restricting brain entry of its substrates. These authors generated mice knocked out for the *Abcb1a* gene and found in part dramatically increased brain concentrations of a range of drugs (e.g., ivermectin, vinblastine, digoxin, cyclosporine A, and loperamide).^1^ These findings led to the expectation that P‐gp would be a rate‐limiting factor for the brain uptake of its substrates and thereby prevent the CNS effects of many drugs. Later data provided evidence that P‐gp may work together with breast cancer resistance protein (BCRP, encoded by the *ABCG2* gene), another ABC transporter abundantly expressed at the BBB, in restricting brain entry of shared P‐gp/BCRP substrates. P‐gp and BCRP are now accepted as functional components of the BBB, and transporter‐mediated efflux of drugs at the BBB is considered a bottleneck in the development of CNS‐acting drugs.

## PET Imaging with Avid P‐gp Substrates

PET imaging with radiolabeled P‐gp substrates has opened up the possibility to directly and noninvasively measure the concentration of these drugs in the human brain.^2^ This made it possible to study the consequences of altered P‐gp function (i.e., by DDIs, age, disease, and genetic polymorphisms) on drug distribution to the brain. Initial probe substrate development for PET focused on compounds that undergo extensive transport by P‐gp at the BBB and which are referred to as “avid” P‐gp substrates in this text (i.e., racemic [^11^C]verapamil, (*R*)‐[^11^C]verapamil, and [^11^C]*N*‐desmethyl‐loperamide).^2^ These compounds were selected based on their high efflux ratios in bidirectional transport assays in P‐gp–overexpressing cells and on the magnitude of the difference in their brain distribution between wild‐type and *Abcb1a/b* ^*(−/−)*^ mice. Their selection was guided by the expectation that high efflux ratios values would translate into a high imaging contrast between situations of normal and altered P‐gp function. As a consequence, these avid P‐gp substrates showed very low brain uptake under conditions when P‐gp was fully functional, due to highly efficient P‐gp–mediated transport at the BBB. However, based on PET experiments with these avid substrates the role of P‐gp in governing the neuropharmacokinetics of its substrates has remained elusive so far. Neither genetic polymorphisms, nor age, nor disease (e.g., drug‐resistant epilepsy, Alzheimer's disease, and depression) were shown to lead to large changes in the brain distribution of these avid substrates, even though a substantial body of evidence had suggested that these conditions were associated with alterations in P‐gp function/expression at the BBB (for a recent review see ref. 3).

## P‐gp–Mediated DDIs at the BBB

Concomitant administration of a transported substrate and inhibitor of P‐gp may lead to partial inhibition of the carrier‐mediated efflux at the BBB, which may result in increases in brain distribution of the substrate drug. This is of concern as P‐gp–mediated DDIs at the BBB may cause CNS adverse effects of drugs, which cannot be predicted from changes in the corresponding plasma pharmacokinetics. PET studies in healthy volunteers showed that the administration of clinically relevant P‐gp inhibitors (cyclosporine A and quinidine) led to only moderate increases in the brain distribution of [^11^C]verapamil.^3^, ^4^ Partly based on these PET results, Kalvass *et al*.^5^ provided an in‐depth analysis of the likelihood of P‐gp–mediated DDIs at the human BBB. The main conclusion of Kalvass was that such DDIs are unlikely to occur as most P‐gp inhibitory drugs used in the clinic will not achieve high enough unbound plasma concentrations to reach sufficiently high P‐gp inhibition levels at the human BBB to translate into clinically relevant alterations in brain distribution of P‐gp substrates. P‐gp is a high‐capacity transporter, which needs to be inhibited by more than 50% to lead to more than twofold changes in brain distribution of its substrates (provided that the substrate is not recognized by another efflux transporter at the BBB).^5^ The conclusions by Kalvass are supported by our own work with (*R*)‐[^11^C]verapamil and [^11^C]*N*‐desmethyl‐loperamide in wild‐type, heterozygous (*Abcb1a/b* ^*(+/−)*^) and homozygous (*Abcb1a/b* ^*(−/−)*^) *Abcb1a/b* knockout mice as models of different P‐gp levels at the mouse BBB.^6^ *Abcb1a/b* ^*(+/−)*^ mice, which have a 50% reduction in P‐gp levels at the BBB as compared with their wild‐type counterparts, showed negligible increases in the brain distribution of (*R*)‐[^11^C]verapamil and [^11^C]*N*‐desmethyl‐loperamide. On the other hand *Abcb1a/b* ^*(−/−)*^ mice, which completely lack P‐gp at the BBB, showed great increases in the brain distribution of these probe substrates relative to wild‐type mice. This highlighted the limited sensitivity of (*R*)‐[^11^C]verapamil and [^11^C]*N*‐desmethyl‐loperamide to detect small (< 50%) changes in P‐gp function at the BBB. A possible way to overcome the limited sensitivity of these probe substrates may be their use under conditions of partial P‐gp inhibition, which, relies on the availability of a P‐gp inhibitor for clinical use.^3^

## Avid vs. Weak P‐gp Substrates for PET

Avid P‐gp substrates employed so far in PET studies confirmed preclinical data regarding the functional barrier role of P‐gp at the BBB.^1^ However, the major part of clinically used CNS‐active drugs possesses a greater passive permeability as compared with these avid P‐gp substrates. Several of these CNS‐active drugs are weak P‐gp substrates, which show appreciable brain distribution despite being transported by P‐gp. In order to better understand the impact of P‐gp on the neuropharmacokinetics of weak P‐gp substrates, it is clearly preferable to use a radiolabeled weak P‐gp substrate for PET studies. Tournier and colleagues have introduced [^11^C]metoclopramide as a weak P‐gp substrate for PET.^7^ Metoclopramide is a peripherally acting antiemetic drug, which shows CNS adverse effects related to inhibition of central dopamine D_2_ receptors, indicating that it distributes to the human brain. Tournier *et al*. demonstrated selectivity of metoclopramide for transport by human P‐gp over BCRP, and lack of brain uptake of radiolabeled metabolites of [^11^C]metoclopramide in rats.^7^ These are two important criteria for an effective P‐gp probe substrate for PET.^2^ Moreover, they demonstrated in rats and nonhuman primates twofold to threefold increases in the brain distribution of [^11^C]metoclopramide following P‐gp inhibition with tariquidar. In their comments to the letter to the editor by Auvity and Tournier, included in this issue, Hsin *et al*.^8^ question the translatability of these results to humans. We agree with these authors that caution is warranted when extrapolating the P‐gp–mediated transport of xenobiotics from preclinical species to humans due to possible species differences in transporter specificities.

**Figure 1:**
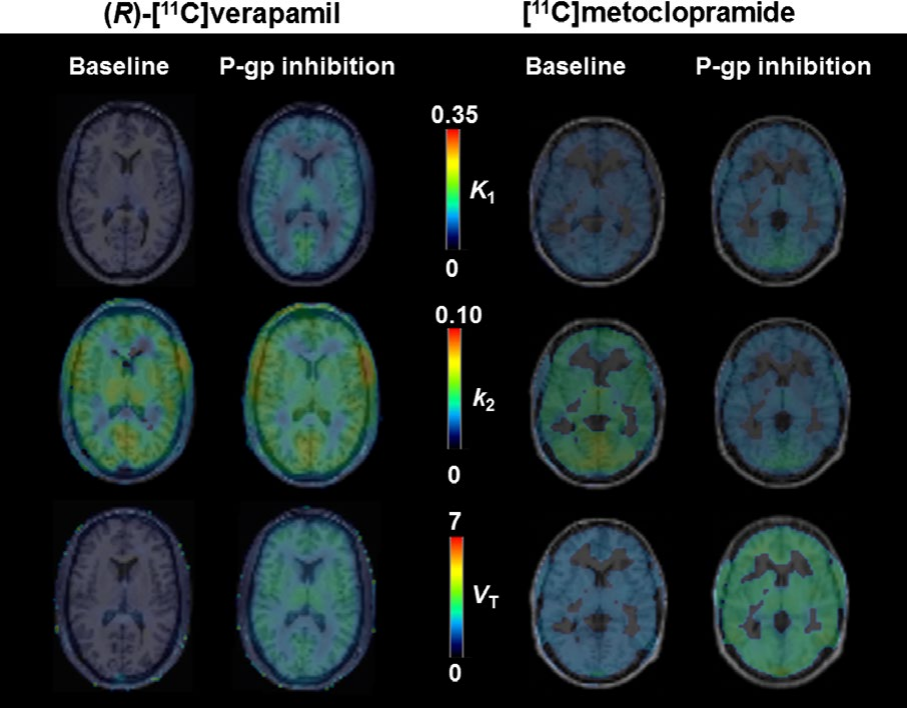
Representative magnetic resonance imaging‐co‐registered parametric positron emission tomography (PET) images in one subject each for (*R*)‐[^11^C]verapamil (left) and [^11^C]metoclopramide (right) at baseline and after P‐glycoprotein (P‐gp) inhibition either with tariquidar (infused during the PET scan at 2.3 mg/kg/hour) or with cyclosporine A (infused during the PET scan at 2.5 mg/kg/hour). In the parametric images, the intensity scales represent the outcome parameters of kinetic modeling (*K*_1_ [mL/(cm^3^.minute)], influx rate constant from plasma into brain; *k*_2_ [1/minute], efflux rate constant from brain into plasma; *V*_T_ [mL/cm^3^], total volume of distribution). At baseline, [^11^C]metoclopramide had a higher brain uptake (*V*_T_, *K*_1_) than (*R*)‐[^11^C]verapamil. For (*R*)‐[^11^C]verapamil, the increase in *V*_T_ (=*K*_1_/*k*_2_) following P‐gp inhibition was caused by an increase in *K*_1_, while for [^11^C]metoclopramide the increase in *V*_T_ was mainly caused by a decrease in *k*_2_. Note that the magnitude of the response to P‐gp inhibition cannot be compared between the two probe substrates due to the higher P‐gp inhibitory potency of tariquidar than that of cyclosporine A. The [^11^C]metoclopramide PET images were originally published in ref. 9, Tournier, N. *et al*. Impact of P‐glycoprotein function on the brain kinetics of the weak substrate ^11^C‐metoclopramide assessed with PET imaging in humans. *J. Nucl. Med*. https://doi.org/10.2967/jnumed.118.219972 (2019). Copyright © SNMMI.

**Figure 2:**
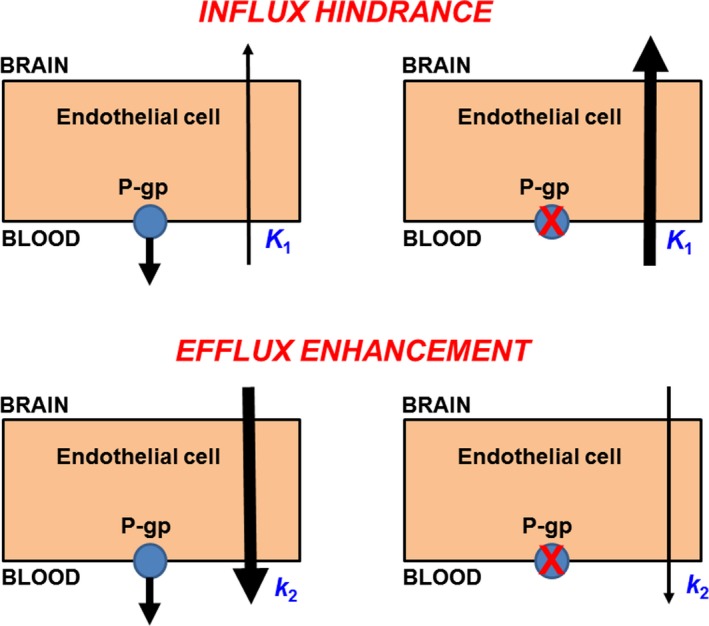
Diagram depicting the principle of influx hindrance and efflux enhancement by P‐glycoprotein (P‐gp) at the blood–brain barrier. For the former, P‐gp inhibition leads to an increase in the influx rate constant from plasma into brain (*K*_1_), while for the latter P‐gp inhibition leads to a decrease in the efflux rate constant from brain into plasma (*k*_2_). It should be noted that both processes can occur at the same time, but efflux enhancement may only be visible when a drug reaches sufficiently high concentration levels in the brain. P‐gp, P‐glycoprotein.

We have characterized the interaction between [^11^C]metoclopramide and the clinically validated P‐gp inhibitor cyclosporine A^4^ in healthy human volunteers with PET imaging.^9^ Our results strongly suggested that [^11^C]metoclopramide is transported by P‐gp at the human BBB as reflected by a 29% increase in its total volume of distribution (*V*_T_) in the brain following cyclosporine A administration. Brain uptake of [^11^C]metoclopramide was substantially higher than that of previously characterized avid P‐gp substrates for PET,^4^ supporting that metoclopramide is a weak P‐gp substrate that can enter the brain despite being transported by P‐gp. Kinetic analysis revealed that the cyclosporine A–induced increase in brain *V*_T_ (=*K*_1_/*k*_2_) was mainly caused by a decrease in the efflux rate constant of [^11^C]metoclopramide from brain into plasma (*k*_2_) rather than by an increase in the influx rate constant from plasma into brain (*K*_1_) (**Figure** 1).^9^ This implies that P‐gp exerts a different impact on the neuropharmacokinetics of the weak P‐gp substrate metoclopramide as compared with previously used avid P‐gp substrates for PET, for which P‐gp inhibition only increased *K*_1_ (**Figure** 1).^4^ P‐gp does not solely contribute to the barrier property of the BBB in limiting influx of drugs from plasma into brain (influx hindrance) but also acts as a detoxifying system to promote clearance of its substrates from the brain (efflux enhancement) (**Figure** 2).^9^, ^10^ Whereas the influx hindrance process effectively keeps drug concentrations in the brain low, efflux enhancement predominantly decreases the half‐life of drugs in the brain.^10^ Focusing on the influx hindrance role of P‐gp may therefore underestimate its importance for the neuropharmacokinetics of drugs and their ability to interact with their respective CNS targets.

Our data thus support the suggestion by Hsin *et al*.^8^ that the CNS adverse effects of metoclopramide may be at least partly controlled by P‐gp function at the human BBB, which raises the possibility of P‐gp–mediated DDIs. The sensitivity of [^11^C]metoclopramide to detect small changes in P‐gp function at the BBB remains to be compared with previously developed PET probe substrates. It can be nonetheless expected that the neuropharmacokinetics of metoclopramide resemble those of a multitude of other CNS‐active weak P‐gp substrates encountered in the clinic (e.g., certain antidepressants, antipsychotics, antiepileptic drugs, and opioids), whose CNS effects may be modulated rather than completely blocked by the action of P‐gp at the BBB. PET data obtained with [^11^C]metoclopramide, and possibly other yet‐to‐be‐developed weak P‐gp substrates for PET, may therefore change our perception of the contribution of P‐gp to the variability in response to CNS‐acting drugs.

## Funding

Austrian Science Fund (FWF) (grant no. KLI 694‐B30).

## Conflict of Interest

The authors declared no competing interests for this work.
